# Suicide prevention in primary health care among socially vulnerable populations: a narrative review

**DOI:** 10.3389/fpubh.2026.1905954

**Published:** 2026-07-13

**Authors:** Lazzat Zhamaliyeva, Akmaral Baspakova, Anara Abitova, Gediminas Merkys, Daiva Bubeliene, Roza Suleimenova, Nurgul Ablakimova

**Affiliations:** 1 Department of General Medical Practice, West Kazakhstan Marat Ospanov Medical University, Aktobe, Kazakhstan; 2 Department of Epidemiology, West Kazakhstan Marat Ospanov Medical University, Aktobe, Kazakhstan; 3 Department of General Hygiene, West Kazakhstan Marat Ospanov Medical University, Aktobe, Kazakhstan; 4 Education Academy, Vytautas Magnus University, Kaunas, Lithuania; 5 Department of Pedagogy, Faculty of Arts and Education, Kauno Kolegija Higher Education Institution, Kaunas, Lithuania; 6 Department of Public Health and Hygiene, Astana Medical University, Astana, Kazakhstan; 7 Department of Pharmacology, Clinical Pharmacology, West Kazakhstan Marat Ospanov Medical University, Aktobe, Kazakhstan

**Keywords:** collaborative care, health inequities, mental health services, primary care, socially vulnerable populations, suicide prevention

## Abstract

**Introduction:**

Suicide remains a major public health challenge worldwide, particularly among socially vulnerable populations who experience disproportionate exposure to social determinants associated with suicide risk and barriers to mental healthcare. Primary health care (PHC) is often the first point of contact with healthcare services and therefore plays a critical role in suicide prevention.

**Methods:**

This narrative review was conducted according to the SANRA guidelines. A structured literature search of PubMed/MEDLINE, Scopus, and Web of Science was performed to identify evidence on suicide prevention interventions implemented in PHC and their applicability to socially vulnerable populations. The review focused on intervention characteristics, implementation approaches, reported outcomes, and challenges relevant to PHC practice.

**Results:**

The reviewed literature indicates that suicide prevention in PHC relies on multiple complementary strategies, including suicide risk screening, collaborative care models, safety planning interventions, brief psychological interventions, telehealth approaches, continuity-of-care programs, and provider training. Evidence suggests that multicomponent and integrated approaches are generally more effective than isolated interventions. Socially vulnerable populations, including people experiencing homelessness, poverty, unemployment, migration, domestic violence, substance use disorders, social isolation, disability, and social marginalization, face additional barriers to accessing mental healthcare and suicide prevention services. Effective interventions frequently combine clinical care with community, social, and support services to address both mental health needs and underlying social determinants of suicide risk.

**Conclusion:**

Suicide prevention in PHC requires integrated, multidisciplinary, and person-centered approaches that extend beyond risk identification alone. Strengthening service integration, continuity of care, provider capacity, and intersectoral collaboration may improve suicide prevention efforts, particularly among socially vulnerable populations. Further research is needed to evaluate implementation strategies and intervention effectiveness in diverse healthcare settings, especially in low- and middle-income countries.

## 1 Introduction

Suicide remains a major global public health challenge and one of the leading causes of preventable mortality worldwide (1). According to the World Health Organization, more than 700,000 people die by suicide annually, with a substantial proportion of deaths occurring in low- and middle-income countries (LMICs) (2). Although global suicide mortality rates have declined over recent decades, suicide continues to impose a considerable social, economic, and healthcare burden, particularly among vulnerable and underserved populations (3, 4).

In recent years, increasing attention has been directed toward the role of social determinants of health in suicidal behavior and mental health outcomes. Socioeconomic instability, poverty, unemployment, social isolation, homelessness, migration, violence exposure, substance use, and limited access to healthcare services have all been associated with elevated suicide risk (5). Socially vulnerable populations are often exposed to multiple overlapping psychosocial stressors while simultaneously experiencing barriers to mental healthcare access, continuity of care, and social support (6, 7). For the purposes of this review, socially vulnerable populations are defined as groups experiencing increased suicide risk and barriers to mental healthcare due to the combined effects of socioeconomic disadvantage, social exclusion, discrimination, exposure to adverse social determinants, and limited access to health and social services. These populations include individuals experiencing homelessness, poverty, unemployment, migration or displacement, domestic violence, substance use disorders, social isolation, disability, and other forms of social marginalization.

Primary health care (PHC) plays a central role in suicide prevention because it often represents the first and most accessible point of contact within the healthcare system. Many individuals experiencing suicidal ideation or mental health difficulties seek care in primary healthcare settings prior to suicide attempts or suicide-related crises (8). PHC professionals are therefore in a unique position to identify suicide risk, provide early intervention, initiate appropriate management, and coordinate referral to specialized mental health services when necessary.

In recent years, multiple suicide prevention interventions have been implemented in primary healthcare settings. These include routine suicide risk screening, collaborative care models, safety planning interventions, psychoeducation, brief psychological interventions, telehealth services, structured follow-up after suicide attempts, and integration of mental health services into PHC practice (8, 9). Educational and training programs for PHC providers have also been developed to improve recognition of suicide risk and enhance confidence in suicide prevention practices. Several interventions have additionally been adapted for vulnerable and underserved populations in order to address social, cultural, and structural barriers influencing access to mental healthcare and continuity of care.

Although the number of studies addressing suicide prevention in PHC has increased substantially, the available evidence remains heterogeneous with respect to intervention models, implementation strategies, healthcare settings, target populations, and reported outcomes. Existing literature includes diverse approaches ranging from screening-based programs to multicomponent collaborative care interventions, making it challenging to provide a comprehensive overview of current practices and evidence, particularly among socially vulnerable groups.

Therefore, the aim of this narrative review is to summarize and discuss contemporary suicide prevention interventions implemented in PHC, with particular attention to intervention characteristics, implementation approaches, outcomes, and applicability among socially vulnerable populations.

## 2 Methods

This narrative review was conducted in accordance with the SANRA (Scale for the Assessment of Narrative Review Articles) guidelines, which provide a framework for improving the methodological quality and transparency of narrative reviews (10).

### 2.1 Literature search strategy

A narrative literature review was conducted to identify and summarize current evidence on suicide prevention interventions implemented in PHC settings and to explore their applicability to socially vulnerable populations. A comprehensive search of the electronic databases PubMed/MEDLINE, Scopus, and Web of Science was performed.

The literature search was conducted in two stages. First, studies evaluating suicide prevention interventions within PHC and PHC settings were identified. Second, an additional targeted search was performed to identify evidence related to suicide prevention among socially vulnerable and underserved populations, including individuals experiencing poverty, unemployment, homelessness, migration, domestic violence, substance use disorders (SUDs), social isolation, disability, and other forms of social disadvantage.

The search strategy combined controlled vocabulary terms and free-text keywords related to suicide prevention, PHC, and vulnerable populations. Search terms included combinations of keywords such as “suicide prevention,” “suicidal ideation,” “suicide attempt,” “self-harm,” “primary health care,” “primary care,” “family medicine,” “collaborative care,” “safety planning,” “mental health integration,” “screening,” “follow-up,” “telehealth,” “socially vulnerable populations,” “poverty,” “unemployment,” “homelessness,” “migration,” “domestic violence,” “substance use disorders,” “social isolation,” “marginalized groups,” and “social determinants of health.” Boolean operators (“AND” and “OR”) were used to combine search terms appropriately. The reference lists of relevant publications were also manually screened to identify additional studies.

### 2.2 Eligibility criteria

Articles were considered eligible if they investigated suicide prevention interventions implemented in PHC; evaluated healthcare-based, community-linked, integrated mental health, digital, telehealth, or public health interventions relevant to suicide prevention; or addressed suicide prevention strategies among socially vulnerable or underserved populations. Studies examining social determinants associated with suicide risk, including poverty, unemployment, homelessness, migration, social isolation, violence exposure, disability, SUDs, and barriers to healthcare access, were also considered eligible.

The review included original peer-reviewed studies (randomized controlled trials, quasi-experimental studies, cohort studies, case–control studies, cross-sectional studies, and qualitative research), as well as systematic reviews, scoping reviews, narrative reviews, meta-analyses, and major evidence-based reports relevant to suicide prevention in PHC settings and vulnerable populations.

Studies were required to report suicide-related outcomes, including suicidal ideation, suicide attempts, self-harm, suicide mortality, help-seeking behavior, treatment engagement, mental health service utilization, or implementation-related outcomes relevant to suicide prevention.

The literature search was conducted between January and May 2026. The final database search was performed on May 1, 2026. Publications available up to this date were considered for inclusion.

### 2.3 Study selection and data extraction

All identified records were imported into EndNote version 21 (Clarivate Analytics, Philadelphia, PA, USA), and duplicate records were removed prior to screening. Titles and abstracts were screened for relevance, followed by full-text assessment of potentially eligible articles.

Articles were excluded if they did not address suicide prevention, PHC, or socially vulnerable populations, or if they did not provide information relevant to the objectives of the review. In cases where multiple publications reported similar findings, priority was given to the most comprehensive and recent sources. Additional relevant studies were identified through manual screening of reference lists of eligible publications. Study selection and eligibility assessment were performed by the authors through discussion and consensus to ensure consistency in the inclusion of relevant literature.

Relevant information from the included studies was extracted narratively and summarized according to study characteristics, target populations, healthcare settings, intervention types, implementation approaches, and reported outcomes. Particular attention was given to commonly described intervention models, including suicide risk screening, collaborative care approaches, safety planning interventions, brief psychological interventions, telehealth-based support, provider education, continuity-of-care strategies, and interventions adapted for socially vulnerable populations.

Due to the narrative design of the review and the broad scope of the topic, both original studies and review articles were included to provide a comprehensive overview of contemporary suicide prevention strategies and their applicability to socially vulnerable populations.

A formal risk-of-bias assessment was not performed because of the narrative design of the review and the heterogeneity of included evidence. However, methodological quality, relevance to PHC practice, and applicability to vulnerable populations were considered during interpretation of the findings.

### 2.4 Data synthesis

The findings were synthesized descriptively using a narrative approach. Included studies were grouped according to major intervention categories, PHC implementation models, and socially vulnerable target populations. The review focused on summarizing the characteristics of interventions, their integration into PHC practice, their applicability to vulnerable groups, and their reported effectiveness in improving suicide-related outcomes.

In addition, gaps in the current literature, implementation challenges, and barriers affecting socially vulnerable populations within PHC settings were identified and discussed.

## 3 Results

### 3.1 Suicide risk screening in primary health care

Suicide risk screening in PHC refers to the systematic identification of individuals who may have suicidal ideation, previous suicidal behavior, self-harm risk, depression, SUDs, or other psychosocial risk factors associated with suicide. In PHC settings, screening is usually performed during routine consultations, mental health assessments, annual check-ups, or visits for depression, anxiety, substance use, chronic pain, social distress, or other high-risk conditions. Screening may be conducted using brief standardized questionnaires, direct clinical questions, electronic health record prompts, or structured triage protocols (11).

The rationale for suicide risk screening in PHC is based on the fact that many individuals at risk of suicide have contact with PHC providers before a suicide-related crisis. In the systematic review by Azizi et al. (12) screening and suicide risk assessment were identified as one of the five major components of suicide prevention strategies in PHC, alongside provider training, management of depression and mental disorders, management of suicide attempters and at-risk cases, and population-level prevention strategies.

In practice, suicide risk screening is often implemented as a stepwise process. First, patients may be screened for depression or psychological distress using brief instruments such as the PHQ-9. If suicidal thoughts are endorsed, particularly through item 9 of the PHQ-9, additional suicide-specific assessment is usually recommended. This may include evaluation of current suicidal ideation, previous suicide attempts, presence of a suicide plan, perceived likelihood of acting on suicidal thoughts, protective factors, access to means, and need for urgent referral or safety planning.

Several instruments have been described for use in PHC or PHC-related settings (Table 1). The PHQ-9, particularly item 9, is frequently used because it is already embedded in depression screening workflows. However, item 9 has limitations because it combines thoughts of death and self-harm and does not directly assess active suicidal intent. Therefore, authors recommend that a positive response should be followed by additional suicide-specific assessment rather than being used as a standalone tool.

**Table 1:** Common suicide risk screening and assessment tools applicable in primary health care.

Tool	Target population	Main purpose	Advantages in PHC	Limitations	Practical recommendation for PHC
PHQ-9 (Item 9)	Adults	Depression and suicidal ideation screening	Brief, widely used in PHC	Limited specificity	Use as an initial screening tool; positive responses should be followed by a suicide-specific assessment.
C-SSRS	Adolescents and adults	Suicide risk assessment	Detailed suicide assessment and triage	Requires training	Suitable for structured suicide risk assessment and referral decision-making.
ASQ	Adolescents and adults	Rapid suicide screening	Very brief and feasible in PHC	Requires follow-up assessment	Recommended for rapid identification of at-risk individuals, followed by a brief suicide safety assessment.
P4 screener	Adults	Suicide risk stratification	Developed for PHC use	Less commonly implemented	Useful for stratifying suicide risk among patients reporting suicidal thoughts.
GDS	Older adults	Screening depressive symptoms and suicide risk	Useful in older adult populations	Not suicide-specific	May be used as an adjunct tool to identify older adults who require further suicide risk assessment.

PHQ-9, Patient Health Questionnaire-9; C-SSRS, Columbia-Suicide Severity Rating Scale; ASQ, Ask Suicide-Screening Questions; P4 Screener, Past Suicide Attempts, Plan, Probability of Completing Suicide, and Preventive Factors Screener; GDS, Geriatric Depression Scale; PHC, Primary Health Care.

The P4 Screener was developed for PHC and specialty medical populations and is triggered when a patient reports thoughts of self-harm. It evaluates four key domains: past suicide attempts, suicide plan, probability of completing suicide, and preventive factors. Based on these domains, it stratifies patients into minimal, lower, or higher suicide risk.

The Ask Suicide-Screening Questions (ASQ) tool is another brief screening instrument. It has been studied in medical and outpatient settings and has high sensitivity and negative predictive value. Following a positive ASQ screen, the toolkit recommends a brief suicide safety assessment to determine whether the patient requires immediate intensive intervention, non-urgent mental health evaluation, or provision of prevention resources. In one large retrospective analysis involving 91,580 patients aged 10–17 years across a safety-net public academic healthcare system, emergency department, and 20 community outpatient clinics, universal ASQ screening identified any suicide risk in 3% of encounters and acute positive screens in 1%.

The Columbia-Suicide Severity Rating Scale (C-SSRS) is also widely described in the literature and has a broad evidence base. The screening version includes two initial questions followed by additional questions depending on the response. In PHC, the C-SSRS may support triage decisions, including no referral, non-urgent behavioral health referral, or urgent behavioral health consultation with safety precautions.

Evidence from PHC studies suggests that screening may improve recognition of suicide risk, but its effectiveness depends on what happens after a positive screen. Saini et al. emphasized that suicide risk screening alone does not reduce suicide attempts when clinical interventions are not implemented. Screening tools may support PHC staff in identifying risk, but they should be combined with provider training, clear referral pathways, safety planning, mental health integration, and follow-up systems.

Large-scale PHC-related interventions have also been reported. In the review by Azizi et al., several studies included population-level or large sample interventions combining screening, provider training, depression management, referral systems, and follow-up. For example, one intervention involving 522,246 individuals included screening and treatment of depressive disorders, provider training, and protocols for identification and treatment, and was associated with improved suicide surveillance and reduced suicides (13). Another adolescent PHC intervention involving 56,352 participants combined staff training, screening, and referral availability and was associated with fewer referrals to emergency departments during the intervention year (14).

However, screening in PHC has important barriers. Bajaj et al. found that although most general practitioners and patients supported screening for suicidal ideation, some felt uncomfortable with such questions (15). Less than half of GPs had received formal training in suicide risk assessment, and reported barriers included time pressure, cultural and language issues, and concern that asking about suicide could negatively affect patients’ mental health.

Communication style is also important. Saini et al. reported that negatively framed or closed questions, such as “You do not feel suicidal, do you?,” may discourage disclosure (16). Clear, direct, and evidence-based wording, including questions similar to PHQ-9 item 9, may improve identification of suicidal ideation in PHC.

Overall, suicide risk screening in PHC should be considered an entry point into a broader suicide prevention pathway that includes assessment, referral, safety planning, follow-up, and access to mental health services (11).

### 3.2 Collaborative care models

In the context of this review, integration refers to the systematic coordination of PHC, mental health services, social care, and community-based resources to provide comprehensive suicide prevention. Levels of integration may range from referral-based collaboration and service coordination to co-located and fully integrated multidisciplinary care models.

Collaborative care models represent a multicomponent approach to suicide prevention in PHC, based on coordination between PHC providers, behavioral health specialists, care managers, nurses, social workers, and, when available, consultant psychiatrists. Unlike isolated screening or referral-based approaches, collaborative care aims to embed mental health expertise into PHC practice through shared treatment planning, systematic follow-up, symptom monitoring, and stepped care. In this model, the PHC provider usually remains responsible for overall patient management, while care managers support follow-up and treatment adherence, and mental health specialists provide consultation for complex or high-risk cases (8).

Across studies, several common components were repeatedly identified. These included regular assessment of depressive symptoms and suicidal ideation, involvement of a care manager, structured communication between PHC and behavioral health providers, psychiatric consultation, treatment adjustment when patients did not improve, and active follow-up rather than passive referral. Grigoroglou et al., in an individual participant data meta-analysis of 28 randomized controlled trials including 11,165 patients, found that collaborative care produced a small but statistically significant reduction in suicidal ideation compared with usual care (17). The effect was stronger when collaborative care included an embedded psychological intervention and appeared more pronounced among older adults.

One of the most frequently cited examples is the PROSPECT trial, a multisite randomized controlled trial conducted in 20 PHC practices (18). This intervention targeted late-life depression and suicide risk by improving recognition and treatment of depression in older adults. The intervention reduced suicidal ideation regardless of depression severity, supporting the role of collaborative care as a suicide prevention strategy in older PHC patients.

More recent reviews also emphasize that collaborative care is not a single intervention, but a framework that allows several suicide prevention components to work together. Spottswood et al. described collaborative suicide prevention in PHC as dependent on the level of behavioral health integration, ranging from informal coordination to co-location and fully integrated care (8). In more integrated settings, practices can use shared protocols, joint monitoring, team-based treatment plans, and clearer role distribution between PHC and behavioral health staff.

There are also implementation-related contradictions. On the one hand, collaborative care is presented as a practical model because it can expand access to mental health support within PHC. On the other hand, its effectiveness depends strongly on available resources, financing, staffing, provider training, and the degree of integration between PHC and mental health services. In poorly integrated systems, more responsibility falls on PHC providers, whereas fully integrated practices can delegate risk assessment, follow-up, and behavioral interventions to embedded mental health professionals (19, 20).

### 3.3 Safety planning interventions

Across the available literature, safety planning interventions were consistently described as one of the most feasible and scalable suicide prevention strategies in PHC settings. Safety planning is generally implemented after identification of suicidal ideation or elevated suicide risk and involves collaborative development of an individualized plan including recognition of warning signs, coping strategies, supportive contacts, professional resources, emergency services, and restriction of access to lethal means. Stanley and Brown originally conceptualized safety planning as a brief intervention intended to mitigate suicide risk during periods of crisis, and this model continues to be widely applied in PHC and emergency settings (21).

Several studies and recent reviews reported that safety planning interventions may reduce acute suicidal crises, improve emotional coping, strengthen treatment engagement, and increase help-seeking behavior, particularly when combined with structured follow-up contacts or continuity-of-care strategies (22, 23).

Across studies, one of the most consistent findings was that safety planning appeared more effective when delivered as part of multicomponent suicide prevention pathways rather than as a standalone intervention. Interventions combining safety planning with follow-up phone calls, caring contacts, referral coordination, or collaborative care models generally demonstrated stronger improvements in suicide-related outcomes compared with isolated safety planning alone (9, 22).

Several authors additionally emphasized the practical advantages of safety planning in PHC settings. Compared with intensive psychiatric interventions, safety planning is relatively brief, low-cost, and adaptable to routine consultations. It may also be delivered by trained PHC providers, nurses, behavioral health staff, or case managers, making it particularly relevant in underserved or resource-limited settings with restricted access to psychiatrists or psychologists (8, 21).

However, evidence regarding the long-term effectiveness of safety planning interventions remains somewhat heterogeneous. While reductions in suicidal ideation and emotional distress were reported relatively consistently, evidence for reductions in suicide attempts and suicide mortality was less uniform across studies (22, 23).

Some studies also suggested that safety planning alone may have limited effectiveness in adolescents or patients with severe psychiatric disorders unless combined with psychotherapy, collaborative care, or intensive follow-up support (24).

Implementation barriers were commonly reported as well. These included insufficient provider training, limited consultation time, uncertainty regarding suicide risk assessment, lack of mental health referral pathways, and reduced adherence among socially vulnerable populations experiencing homelessness, SUDs, social isolation, or unstable living conditions (8, 15).

### 3.4 Brief psychological interventions

Brief psychological interventions for suicide prevention include short CBT-based approaches, motivational interviewing, problem-solving therapy, and psychoeducation. Across studies, these interventions are generally aimed at reducing acute psychological distress, improving coping skills, increasing treatment engagement, and strengthening patients’ ability to manage suicidal thoughts in non-specialized settings such as PHC (25).

Younesi et al. (9) identified only a small number of PHC studies and reported that motivational interviewing, structured follow-up, safety planning, and collaborative care were the main brief interventions evaluated for suicidal ideation in PHC.

CBT-based brief interventions appear promising, especially when they are suicide-focused and structured. A recent randomized clinical trial showed that brief cognitive behavioral therapy delivered via video telehealth reduced suicide attempts among adults with recent suicidal thoughts or behaviors, suggesting that brief CBT can be adapted beyond traditional face-to-face specialist settings (26).

Problem-solving therapy has also been associated with reductions in suicidal ideation in older adults with depression and executive dysfunction, supporting its potential relevance for PHC populations where depression, chronic illness, and psychosocial stressors often coexist (27).

However, the evidence base for brief psychological interventions in PHC remains relatively limited compared with other intervention categories. Most studies evaluate suicidal ideation or psychological distress rather than suicide attempts or suicide mortality, and considerable heterogeneity exists regarding intervention content, duration, provider training, and outcome measurement. These factors complicate direct comparisons across studies and limit conclusions regarding long-term effectiveness.

### 3.5 Telehealth and digital interventions

Telehealth and digital interventions include telepsychiatry, video consultations, telephone support, online follow-up, mobile applications, digital safety plans, and web-based self-help tools. Across studies, the most consistent advantage is improved access to care, especially for rural, remote, underserved, or socially vulnerable populations who may face barriers to in-person mental health services (28).

Recent reviews suggest that telehealth-based suicide prevention interventions may reduce suicidal ideation when they include structured clinical components such as crisis response planning, safety planning, or clinician-guided follow-up. Digital suicide prevention tools, including mobile applications and web-based interventions, are increasingly studied, but the evidence remains mixed and depends strongly on whether the intervention is guided, monitored, and linked to clinical care (29).

The strongest results appear to come from hybrid or clinician-supported models rather than fully self-guided applications. For example, video-delivered brief CBT showed effectiveness in reducing suicide attempts, supporting the idea that telehealth can be clinically meaningful when it preserves professional contact and structured intervention content (26).

Digital interventions also have substantial limitations. Fully self-guided mobile applications are frequently associated with low adherence, high dropout rates, reduced long-term engagement, and difficulties in adequate safety monitoring. In addition, digital exclusion remains an important concern among socially vulnerable populations, including individuals experiencing poverty, unstable housing, limited digital literacy, inadequate internet access, disability, or language-related barriers. Consequently, telehealth and digital approaches should be considered complementary components of suicide prevention in PHC rather than substitutes for direct clinical assessment, ongoing monitoring, and continuity of care.

### 3.6 Follow-up and continuity-of-care strategies

Follow-up and continuity-of-care strategies include post-attempt follow-up, telephone calls, caring contacts, appointment coordination, active outreach, and continuity after emergency department or hospital discharge. In the context of this review, continuity of care is understood as the degree to which healthcare services are coordinated, connected, and experienced as consistent over time. In suicide prevention, continuity of care includes timely follow-up after suicidal crises, coordination between PHC, mental health, and social services, long-term patient engagement, and relational continuity with healthcare providers. Evidence consistently identifies continuity-of-care interventions as an effective component of suicide prevention, particularly because they address the vulnerable transition period between acute crisis treatment and reintegration into community-based care (30).

Continuity of care is repeatedly described as central to suicide prevention because it helps maintain patient contact, reduce isolation, improve engagement, and connect patients with ongoing mental health support. Contemporary reviews emphasize that continuity may include timely follow-up, active linkage, shared care planning, monitoring, and coordination between emergency, PHC, and mental health services (6).

Evidence from follow-up studies suggests that telephone follow-up and brief contact interventions can reduce repeated suicide attempts after discharge from emergency or psychiatric services. Some controlled studies reported that very early telephone follow-up was associated with reduced recurrence of suicide attempts (30). Recent meta-analytic evidence also supports the role of brief interventions and follow-up contacts as scalable approaches after suicide attempts (31). Nevertheless, the optimal frequency, duration, and mode of follow-up remain uncertain. Furthermore, evidence regarding effects on suicide mortality is less robust than evidence for reductions in repeat suicide attempts and improvements in treatment engagement.

The impact of these interventions on suicide mortality is more difficult to evaluate because suicide deaths are relatively infrequent outcomes and typically require large populations and prolonged observation periods to demonstrate measurable effects. Additional challenges arise when interventions are implemented among socially vulnerable populations, where continuity of care may be disrupted by unstable housing conditions, migration, limited transportation, changing contact information, communication barriers, or reduced trust in healthcare systems.

### 3.7 Education and training of PHC providers

Several studies have demonstrated that stigma, discomfort, and lack of confidence among PHC providers remain important barriers to effective mental health and suicide prevention care. PHC professionals may feel uncertain about how to assess mental health conditions and suicide risk, fear that discussing suicidal thoughts could worsen emotional distress, or experience anxiety regarding management of high-risk patients. Negative attitudes toward mental illness, insufficient mental health training, limited confidence in communication skills, and concerns about damaging the therapeutic relationship may further contribute to underrecognition of depression, psychological distress, and suicidal ideation in PHC settings (32). Bajaj et al. (15) reported that although many general practitioners supported suicide screening, some remained uncomfortable discussing suicidal thoughts with patients, and less than half had received formal training in suicide risk assessment. Similarly, Saini et al. (16) noted that PHC providers may avoid direct suicide-related questions or use negatively framed language because of discomfort, stigma, or uncertainty regarding suicide assessment.

Consequently, education and training of PHC providers have become central components of contemporary suicide prevention strategies. Across studies, training interventions generally aimed to improve suicide risk recognition, communication skills, confidence in discussing suicidal thoughts, referral practices, crisis response, and reduction of stigma toward suicidal patients.

One of the most consistent findings across the literature is that suicide prevention training improves provider-level outcomes, particularly knowledge, confidence, self-efficacy, and willingness to ask directly about suicidal ideation. Recent systematic review and meta-analysis evidence demonstrated that gatekeeper training programs significantly improved suicide-related knowledge, attitudes, and intervention preparedness among healthcare workers and community gatekeepers (4). Similarly, training interventions among nurses and PHC-related staff were associated with improvements in confidence and perceived ability to recognize suicide risk and respond appropriately (33).

Several countries have implemented national or large-scale suicide prevention training strategies integrated into PHC systems. In the United Kingdom, suicide prevention guidance emphasizes suicide risk assessment training, safety planning, continuity of care, and collaboration between PHC and mental health services as essential components of suicide prevention practice (34). In Australia, the National Suicide Prevention Strategy and LifeSpan model incorporated community education, gatekeeper training, PHC engagement, and coordinated care pathways across multiple regions. Evaluations suggested improvements in awareness, referral coordination, and local suicide prevention capacity (35).

The Zero Suicide framework, implemented in the United States and adapted in several healthcare systems internationally, also emphasizes workforce education and organizational culture change. This approach includes systematic suicide screening, staff competency training, standardized pathways, and continuous quality improvement within healthcare services (36).

Across studies, the most effective training models appeared to be interactive and repeated rather than one-time educational sessions. Programs involving role-play, simulation, case discussions, multidisciplinary learning, and refresher training generally produced stronger improvements in provider preparedness and communication skills compared with passive lecture-based education alone (37).

Despite these positive findings, evidence regarding the effect of provider training on patient-level outcomes remains less consistent. While improvements in knowledge, attitudes, and confidence were repeatedly demonstrated, reductions in suicide attempts and suicide mortality were less clearly established across studies. Some authors suggested that training alone is insufficient unless accompanied by organizational support, standardized protocols, referral pathways, mental health integration, and continuity-of-care systems (4, 38).

### 3.8 Suicide prevention among socially vulnerable populations

Socially vulnerable populations were consistently described as groups with a higher burden of suicide-related risk factors, including poverty, unemployment, unstable housing, violence exposure, migration-related stress, disability, SUDs, social isolation, stigma, and limited access to mental health care. These factors rarely occur in isolation and often overlap, creating cumulative psychosocial vulnerability and barriers to timely help-seeking. Recent reviews on social determinants of mental health and suicide emphasize that effective prevention should address both clinical risks and broader social determinants such as housing, income insecurity, discrimination, social isolation, and access to care.

#### 3.8.1 Homeless individuals

People experiencing homelessness represent one of the most vulnerable populations for suicide prevention because homelessness is closely linked to multiple interacting social and health-related determinants, including mental disorders, SUDs, trauma exposure, chronic physical illness, poverty, social exclusion, and limited access to healthcare services. Recent evidence indicates that mental health disorders are highly prevalent among people experiencing homelessness. A 2024 systematic review and meta-analysis including 85 studies and 48,414 participants reported that the current prevalence of mental health disorders among homeless individuals was 67% (95% CI 55–77%), while lifetime prevalence reached 77% (95% CI 61–88%) (39).

Depression represents one of the most common psychiatric conditions in this population. A systematic review and meta-analysis found a pooled prevalence of depressive symptoms of 46.7% and major depressive disorder of 26.2% among homeless individuals, substantially exceeding estimates reported for the general population (40).

Suicide risk is also markedly elevated among people experiencing homelessness. A recent nationwide cohort study published in *The Lancet Public Health* reported that within 10 years of first contact with homeless shelters, approximately 1.3% of men and 0.9% of women died by suicide, while nearly one in ten individuals experienced a suicide attempt requiring healthcare contact (41). Studies further suggest that suicidal behavior among homeless populations is strongly influenced by co-occurring SUDs, trauma exposure, victimization, hopelessness, and social isolation. A recent review highlighted a consistent association between psychoactive substance use and increased suicidal ideation and suicidal behavior among homeless individuals, particularly among those experiencing multiple psychosocial vulnerabilities (42, 43).

Evidence from the At Home/Chez Soi study demonstrated that homeless adults with mental disorders had a substantial burden of suicidal ideation and previous suicide attempts, highlighting the importance of targeted public health interventions in this population. The Housing First model is one of the most extensively studied interventions in this group. In the At Home/Chez Soi randomized trial, participants allocated to Housing First spent approximately 73% of their time in stable housing during the first year, compared with 30% among those receiving treatment as usual (44).

Despite these positive housing and social outcomes, suicide-specific effects were less conclusive. Aquin et al. (44) found no clear evidence that Housing First was superior to treatment as usual in reducing suicidal ideation or suicide attempts among homeless adults with mental disorders.

#### 3.8.2 Low-income and unemployed populations

Low-income and unemployed individuals constitute a socially vulnerable population with an elevated risk of suicidal behavior because financial insecurity, unemployment, debt, housing instability, and social deprivation are strongly associated with psychological distress, depression, hopelessness, and reduced access to healthcare services. Unemployment has consistently been identified as one of the most important social determinants associated with suicide risk, particularly during periods of economic instability and widening social inequalities. The relationship between poverty and suicide is complex and appears to be mediated through multiple pathways, including chronic stress, financial strain, food insecurity, social isolation, reduced self-esteem, family conflict, and barriers to accessing healthcare and social support services (45, 46).

Evidence from large population-based studies has demonstrated a strong association between unemployment and adverse mental health outcomes. A meta-analysis by Paul and Moser reported significantly higher levels of depression, anxiety, psychological distress, and reduced well-being among unemployed individuals compared with employed populations (47). Similarly, Norström and Grönqvist (46) found that increases in unemployment rates were associated with increased suicide mortality, particularly among working-age adults, highlighting the importance of macroeconomic conditions as determinants of population mental health. Additional evidence suggests that financial hardship and income insecurity may contribute to suicidal ideation independently of psychiatric disorders, emphasizing the role of broader social and economic factors in suicide prevention (48).

Given the important contribution of socioeconomic factors to suicide risk, several authors have recommended supplementing standard suicide risk assessment tools, such as the PHQ-9 and C-SSRS, with screening instruments that assess social determinants of health (49). Commonly used tools include the Protocol for Responding to and Assessing Patients’ Assets, Risks, and Experiences (PRAPARE) and the (50) Screening Tool, which evaluate domains such as financial strain, food insecurity, housing instability, transportation barriers, employment status, and social support (51). These instruments may help identify unmet social needs that contribute to psychological distress and potentially increase suicide risk among economically vulnerable populations.

One of the most frequently discussed approaches in recent years is social prescribing. This model allows PHC providers to refer patients to non-clinical community resources such as peer-support groups, volunteering opportunities, educational programs, financial advice services, physical activity programs, and social activities (52). Several studies have reported improvements in psychological well-being, social connectedness, loneliness, and depressive symptoms following social prescribing interventions, particularly among socially disadvantaged populations (53, 54). By addressing social isolation and unmet social needs, these interventions may indirectly reduce factors associated with suicidal behavior.

Several interventions have also focused on integrating welfare and financial support within healthcare settings. For example, co-located welfare advice services in PHC have been associated with improvements in financial security, reduced stress related to debt and benefits, and better mental well-being among socioeconomically disadvantaged patients (55, 56). Although these programs were not designed specifically as suicide prevention interventions, they address several established determinants of suicidal behavior, including financial strain, social exclusion, and barriers to accessing support services (57, 58).

Employment-support interventions have also demonstrated promising results. Programs combining vocational rehabilitation, employment assistance, psychological support, and case management have been associated with improvements in self-esteem, social functioning, and mental health outcomes among unemployed individuals (59). In some studies, successful re-employment was associated with reductions in depressive symptoms and psychological distress, suggesting that restoring economic participation may contribute to improved mental well-being and potentially reduce suicide risk (60, 61). The effectiveness of employment-related interventions may partly reflect the broader psychological and social benefits of employment. Beyond income generation, employment may enhance social connectedness, daily structure, self-efficacy, and perceived purpose in life, all of which have been identified as protective factors for mental health.

Most studies evaluate intermediate outcomes such as depression, anxiety, quality of life, social participation, and healthcare utilization rather than suicide-specific endpoints. Furthermore, the effectiveness of interventions may depend on broader economic conditions, labour market opportunities, social protection systems, and welfare policies that extend beyond the healthcare sector (62).

#### 3.8.3 Migrants and displaced populations

Migrants, refugees, asylum seekers, and forcibly displaced populations represent a heterogeneous but particularly vulnerable group for suicide prevention. Migration-related stressors, including forced displacement, exposure to conflict and violence, family separation, acculturation difficulties, discrimination, language barriers, legal insecurity, and limited access to healthcare services, may substantially increase the risk of mental health disorders and suicidal behavior. Importantly, suicide risk among migrants is not uniform and varies according to migration pathway, country of origin, host-country conditions, socioeconomic status, and previous exposure to trauma.

Mental health disorders are highly prevalent among displaced populations. A systematic review and meta-analysis reported pooled prevalence estimates of approximately 31% for post-traumatic stress disorder (PTSD), 31% for depression, and 11% for anxiety disorders among refugees and conflict-affected populations (63). Similarly, WHO has identified refugees and migrants as populations at increased risk of depression, anxiety, PTSD, and psychological distress due to cumulative exposure to pre-migration, migration, and post-migration stressors (64).

Several studies have demonstrated elevated rates of suicidal ideation and suicidal behavior among refugees and asylum seekers. Although reported prevalence estimates vary substantially across studies and settings, suicidal ideation appears to be consistently associated with cumulative trauma exposure, social exclusion, discrimination, and post-migration stressors, particularly among asylum seekers facing prolonged legal uncertainty (65, 66).

Given the central role of trauma and migration-related stressors, suicide risk assessment in migrant populations often extends beyond standard suicide screening tools. In addition to instruments such as the PHQ-9 and C-SSRS, studies frequently employ the Harvard Trauma Questionnaire (HTQ), PTSD Checklist (PCL-5), Refugee Health Screener (RHS-13), and other culturally adapted instruments designed to assess trauma exposure, psychological distress, and migration-related vulnerabilities (67–70).

Several interventions have been developed specifically for migrant and refugee populations. These include culturally adapted cognitive behavioral therapy, community outreach programs, peer-support interventions, interpreter-supported mental healthcare, trauma-informed services, and digital mental health interventions. Evidence suggests that culturally adapted interventions may improve treatment engagement, acceptability, and mental health outcomes by addressing language barriers, cultural beliefs, stigma, and differences in help-seeking behavior (71, 72).

Digital and telehealth-based interventions have received increasing attention in recent years. For example, guided online interventions specifically adapted for migrant populations have demonstrated feasibility and acceptability for reducing psychological distress and suicidal ideation, particularly among individuals who face barriers to accessing conventional mental healthcare services (73). Community-based interventions involving peer navigators and cultural mediators have also been associated with improved service utilization and greater trust in healthcare providers.

Despite these promising findings, evidence regarding suicide-specific outcomes remains limited. Most intervention studies focus on PTSD symptoms, depression, anxiety, psychological distress, or healthcare engagement rather than suicide attempts or suicide mortality. Furthermore, the effectiveness of interventions may vary considerably according to migration experiences, cultural context, legal status, and availability of social support.

#### 3.8.4 Survivors of domestic violence

Survivors of domestic violence represent a particularly vulnerable population for suicide prevention because exposure to physical, sexual, psychological, or economic abuse is strongly associated with depression, anxiety, PTSD, SUDs, social isolation, and suicidal behavior. The relationship between intimate partner violence (IPV) and suicide risk has been consistently demonstrated across different settings and populations, with survivors frequently experiencing multiple overlapping psychosocial stressors that may contribute to feelings of hopelessness, entrapment, and reduced access to support services (74).

A recent systematic review and meta-analysis reported that individuals exposed to intimate partner violence had significantly higher odds of suicidal ideation, suicide attempts, and self-harm compared with those who had not experienced violence (75). The risk appears to be particularly elevated among survivors exposed to repeated violence, coercive control, sexual abuse, and concurrent mental health disorders. In addition, social isolation, financial dependence, housing insecurity, and fear of further victimization may further increase vulnerability and reduce help-seeking behavior.

Given the strong association between violence exposure and suicide risk, several organizations recommend routine enquiry or targeted screening for intimate partner violence in healthcare settings. In addition to standard suicide risk assessment tools such as the PHQ-9 and C-SSRS, commonly used instruments include the Hurt, Insult, Threaten, Scream (HITS) tool, the Woman Abuse Screening Tool (WAST), and the Danger Assessment Scale, which help identify individuals at risk of ongoing violence and severe harm (76).

Several interventions have been developed to support survivors of domestic violence. These include trauma-informed care, advocacy and case-management programs, safety planning interventions, psychosocial counselling, referral to legal and social services, and integrated healthcare-community responses. Evidence suggests that advocacy interventions linking survivors with community resources may improve mental health outcomes, safety behaviors, and access to support services, particularly when delivered as part of coordinated multidisciplinary care (77).

Safety planning has emerged as one of the most relevant interventions for this population. Individualized safety plans may help survivors identify warning signs of escalating violence, develop emergency coping strategies, strengthen social support networks, and facilitate timely access to crisis services (78–80).

Digital and mobile health interventions have also received increasing attention. Online safety-planning tools, web-based advocacy programs, and mobile applications have demonstrated potential for improving access to support among individuals who may be unable or unwilling to seek face-to-face services (81, 82). However, concerns regarding confidentiality, partner surveillance, digital literacy, and unequal access to technology remain important challenges for implementation.

Despite encouraging findings, most intervention studies focus on mental health outcomes, safety behaviors, PTSD symptoms, depression, or service utilization rather than suicide-specific outcomes. Furthermore, survivors of domestic violence often face multiple structural barriers, including economic dependence, housing instability, stigma, and limited access to legal protection, which may reduce the effectiveness of isolated healthcare interventions.

From a public health perspective, suicide prevention among survivors of domestic violence requires trauma-informed, survivor-centred, and multidisciplinary approaches that integrate mental healthcare with social, legal, housing, and community support services. Addressing both violence exposure and its broader social consequences may be essential for reducing long-term suicide risk in this population.

#### 3.8.5 Individuals with substance use disorders

Individuals with SUDs represent one of the highest-risk groups for suicidal behavior. The relationship between substance use and suicide is multifactorial and involves biological, psychological, and social mechanisms. Alcohol and drug use may increase impulsivity, impair judgment, exacerbate psychiatric symptoms, reduce problem-solving abilities, and increase exposure to adverse social circumstances, including homelessness, unemployment, social isolation, and victimization. Moreover, SUDs frequently co-occur with depression, anxiety, trauma-related disorders, and other psychiatric conditions that independently increase suicide risk (83).

A recent umbrella review reported that individuals with SUDs experience substantially higher rates of suicidal ideation, suicide attempts, and suicide mortality compared with the general population. The risk appears particularly elevated among individuals with opioid use disorder, polysubstance use, co-occurring mental illness, and previous suicide attempts. Alcohol use disorder has been consistently identified as one of the strongest modifiable risk factors for suicide across different populations and healthcare settings (84).

Given the complex relationship between substance use and suicidality, assessment typically extends beyond standard suicide screening instruments. In addition to tools such as the PHQ-9 and C-SSRS, clinicians frequently use instruments including the Alcohol Use Disorders Identification Test (AUDIT), Drug Abuse Screening Test (DAST-10), Alcohol, Smoking and Substance Involvement Screening Test (ASSIST), and addiction-specific clinical assessments to evaluate substance use severity and related psychosocial risks (85–87).

Several interventions have been developed to address suicide risk among individuals with SUDs. These include integrated treatment models, motivational interviewing, cognitive behavioral therapy, medication-assisted treatment, collaborative care approaches, safety planning, and continuity-of-care interventions. Evidence suggests that integrated treatment addressing both substance use and mental health conditions is more effective than fragmented or parallel models of care, particularly among individuals with co-occurring psychiatric disorders (88, 89).

Safety planning interventions have also shown promise in addiction treatment settings. By identifying personal warning signs, coping strategies, supportive contacts, and crisis resources, safety planning may help reduce acute suicide risk while improving treatment engagement. In addition, several studies have demonstrated that incorporating suicide prevention modules within substance use treatment programs can improve suicide-related knowledge, increase help-seeking behavior, and strengthen connections to mental healthcare services (21).

One important challenge is that suicide risk among individuals with SUDs is highly dynamic and may fluctuate during periods of intoxication, withdrawal, relapse, treatment initiation, or major life transitions (90). Consequently, timely identification of acute risk and sustained engagement in care may be as important as the intervention itself.

The available evidence suggests that suicide prevention should be embedded throughout the continuum of addiction care rather than delivered as a separate service. Routine suicide risk assessment, safety planning, coordinated management of co-occurring psychiatric disorders, and proactive follow-up during high-risk periods may represent key components of effective prevention strategies (91). Future research should focus on identifying interventions capable of reducing suicide attempts and suicide mortality while maintaining long-term engagement among individuals with SUDs.

#### 3.8.6 Socially isolated individuals and people with disabilities

Social isolation and disability have emerged as important determinants of suicide risk in contemporary public health research (92). Individuals experiencing loneliness, limited social participation, reduced community engagement, chronic illness, disability-related stigma, functional impairment, or dependence on caregivers may face increased psychological distress and reduced access to social and healthcare resources. Unlike many traditional clinical risk factors, social isolation often develops gradually and may remain undetected in routine healthcare encounters (93).

Recent evidence indicates that loneliness and social isolation are independently associated with depression, anxiety, suicidal ideation, and suicide mortality (94). A growing body of research suggests that perceived loneliness may be as important as objective social isolation in determining mental health outcomes. Similarly, people with disabilities experience elevated rates of mental health disorders, social exclusion, unemployment, poverty, and barriers to healthcare, all of which may contribute to increased suicide risk (95).

Assessment in these populations often includes evaluation of social connectedness, social support, functional status, disability-related needs, and quality of life in addition to standard suicide risk screening tools. Commonly used instruments include the UCLA Loneliness Scale, Lubben Social Network Scale, WHO Disability Assessment Schedule (WHODAS 2.0), and disability-specific functional assessments (96–98).

Several interventions have been developed to address social isolation and its mental health consequences. Social prescribing has emerged as one of the most widely discussed approaches in PHC (99). Through referral to community organizations, volunteering opportunities, support groups, educational activities, and recreational programs, social prescribing aims to strengthen social connectedness and reduce loneliness (100). Multiple studies have reported improvements in well-being, social participation, loneliness, and depressive symptoms following social prescribing interventions, particularly among socially isolated individuals and older adults (101).

Community-based peer-support programs, telephone outreach services, befriending interventions, and group-based psychosocial activities have also demonstrated positive effects on social engagement and psychological well-being (102, 103). Among people with disabilities, integrated models that combine rehabilitation services, mental healthcare, social support, and caregiver involvement appear particularly beneficial (104, 105).

A notable challenge in these populations is that loneliness and social isolation frequently remain unrecognized despite regular contact with healthcare services. Unlike many clinical risk factors, social disconnection may develop gradually and persist for prolonged periods before becoming visible to healthcare providers. Consequently, interventions aimed at strengthening social participation and community engagement may serve not only therapeutic but also preventive functions.

#### 3.8.7 Marginalized and underserved populations

An important challenge in suicide prevention is that some individuals remain underserved by healthcare systems despite having multiple risk factors for suicidal behavior. These populations often include people who experience social exclusion, discrimination, stigma, distrust of institutions, or practical barriers to healthcare access (106). Rather than being defined by a single characteristic, marginalization frequently results from the accumulation of disadvantages across multiple domains, including socioeconomic status, education, housing, employment, legal status, and healthcare access (107).

Individuals from marginalized and underserved communities often encounter substantial obstacles when seeking mental healthcare. These barriers may include financial constraints, transportation difficulties, language and cultural challenges, stigma, concerns about discrimination, and previous negative experiences with healthcare systems, all of which can reduce service utilization and contribute to unmet mental health needs and discontinuity of care (108).

A recurring finding across the literature is that healthcare accessibility may be as important as the intervention itself. Consequently, many successful programs have focused not only on mental health treatment but also on reducing barriers to care. Community outreach initiatives, mobile health services, peer navigator programs, culturally adapted care pathways, and integrated PHC models have all been associated with improved healthcare engagement among underserved populations (109, 110).

Particularly promising results have been reported for peer-led and community-based approaches. Individuals with lived experience or strong community connections may help bridge gaps between healthcare services and populations that traditionally underutilize formal care.

Across socially vulnerable populations, several common mechanisms contribute to elevated suicide risk, including poverty, social exclusion, stigma, discrimination, unstable housing, limited social support, and barriers to healthcare access. These factors frequently coexist and interact, creating cumulative vulnerability that cannot be adequately addressed through isolated clinical interventions alone. Across populations, the most consistently reported implementation challenges include fragmented services, poor continuity of care, workforce limitations, and difficulties maintaining long-term engagement with healthcare and social support systems. Consequently, effective suicide prevention for socially vulnerable populations is likely to require integrated approaches that combine clinical care with interventions targeting broader social determinants of health.

### 3.9 Barriers to implementation of suicide prevention interventions in primary health care

Despite growing evidence supporting suicide prevention interventions in PHC, implementation remains challenging across many healthcare systems. One of the most frequently reported barriers is insufficient workforce capacity. PHC providers often face high patient volumes, competing clinical priorities, and limited time for comprehensive mental health assessment, making routine suicide risk screening and follow-up difficult to implement consistently (8, 15, 16).

Some providers report uncertainty regarding suicide risk assessment, safety planning, and management of high-risk patients, while others express concerns about increasing workload or potential legal responsibility. Mental health stigma among healthcare professionals may further contribute to underrecognition of suicidal ideation and reduced engagement in suicide prevention activities (32).

Limited availability of referral pathways represents another major challenge. Even when suicide risk is identified, PHC providers may encounter difficulties accessing specialist mental health services, crisis intervention teams, addiction treatment programs, or community support resources. Fragmentation between PHC, mental health services, social care, and community organizations may result in delayed referrals, interrupted continuity of care, and loss of follow-up for high-risk individuals (6).

Financial constraints and insufficient organizational support also influence implementation. Several studies have reported that collaborative care models, integrated mental healthcare, and continuity-of-care programs require sustainable funding, dedicated personnel, and institutional commitment. In resource-limited settings, competing healthcare priorities may limit the capacity to implement comprehensive suicide prevention strategies (111).

Additional challenges are frequently observed in rural and remote areas. Geographic isolation, shortages of mental health professionals, transportation barriers, limited availability of specialist services, and concerns regarding confidentiality within small communities may reduce access to suicide prevention services and contribute to unmet mental health needs (112).

**Figure 1:**
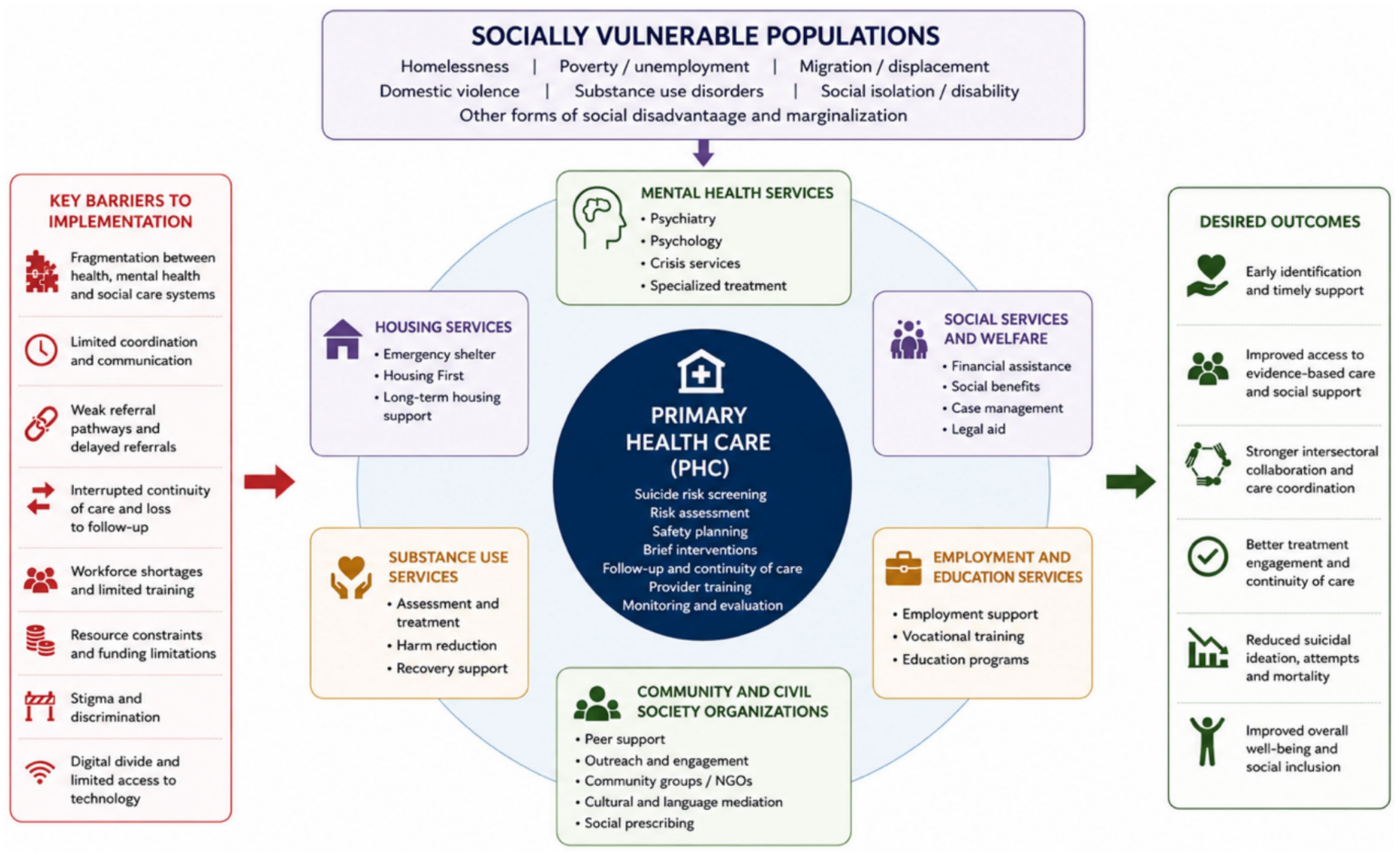
Intersectoral approach to suicide prevention in primary health care for socially vulnerable populations (authors’ conceptual framework based on the reviewed literature created with ChatGPT).

Collectively, these findings suggest that successful implementation of suicide prevention interventions in primary healthcare depends not only on the effectiveness of individual interventions but also on broader health system capacity, workforce development, service integration, and equitable access to care. As illustrated in Figure 1, effective suicide prevention for socially vulnerable populations requires coordinated collaboration between primary healthcare providers, mental health services, social care, community organizations, and other support sectors. Fragmentation of these services may undermine continuity of care and reduce the effectiveness of otherwise evidence-based interventions. A summary of the major intervention categories, target populations, reported outcomes, levels of evidence, and key implementation considerations is presented in Table 2.

**Table 2:** Summary of suicide prevention interventions in primary health care and their applicability to socially vulnerable populations.

Intervention category	Target population	Main reported outcomes	Level of evidence	Key implementation considerations
Suicide risk screening	General PHC population; adolescents; adults; older adults	Improved identification of suicidal ideation and suicide risk; increased referral to mental health services	Systematic reviews, primary studies	Most effective when linked to referral pathways, safety planning, and follow-up care; provider training required
Collaborative care models	Adults with depression; older adults; patients with chronic conditions	Reduced suicidal ideation; improved depression outcomes; increased treatment engagement	Meta-analyses, RCTs, systematic reviews	Requires integration of PHC and mental health services, care managers, multidisciplinary teams, and sustainable funding
Safety planning interventions	Individuals with suicidal ideation or elevated suicide risk	Improved coping, crisis management, treatment engagement, and help-seeking behavior	Systematic reviews, primary studies	Low-cost and feasible in PHC; most effective when combined with follow-up contacts and referral support
Brief psychological interventions	Adults with suicidal ideation; patients with depression and psychosocial distress	Reduced suicidal ideation; improved coping skills and psychological well-being	RCTs, systematic reviews	Requires trained personnel; evidence stronger for ideation than for suicide mortality
Telehealth and digital interventions	Rural, remote, underserved, and socially vulnerable populations	Improved access to care; reduced psychological distress and suicidal ideation in some studies	Systematic reviews, primary studies	Digital exclusion, low adherence, and safety monitoring remain challenges; clinician-supported approaches appear most effective
Follow-up and continuity-of-care strategies	Individuals after suicidal crises, emergency department visits, or hospital discharge	Reduced repeat suicide attempts; improved treatment engagement and continuity of care	Meta-analyses, systematic reviews, primary studies	Early follow-up and active outreach are critical; continuity may be difficult among socially vulnerable populations
Education and training of PHC providers	PHC physicians, nurses, and other healthcare professionals	Improved knowledge, confidence, attitudes, and preparedness for suicide prevention	Systematic reviews, meta-analyses	Effects on provider outcomes are well established; evidence for effects on suicide mortality remains limited
Social prescribing and community linkage	Socially isolated, low-income, unemployed, and marginalized populations	Improved social connectedness, well-being, and mental health outcomes	Primary studies, systematic reviews	Requires community resources and cross-sector collaboration; suicide-specific outcomes rarely evaluated
Housing, welfare, and social support interventions	Homeless individuals; socioeconomically disadvantaged populations	Improved housing stability, access to services, and psychosocial outcomes	RCTs, cohort studies, systematic reviews	Address social determinants of suicide risk; evidence for direct effects on suicide outcomes remains limited
Culturally adapted and trauma-informed interventions	Culturally adapted and trauma-informed interventions	Improved treatment engagement, mental health outcomes, and service utilization	Systematic reviews, primary studies	Adaptation to cultural context, language, and trauma history is essential for effectiveness

PHC, primary health care; RCTs, randomized controlled trials.

#### 3.9.1 Gaps in current evidence

Although the literature on suicide prevention in PHC has expanded considerably, several important evidence gaps remain. First, most studies have been conducted in high-income countries, particularly in North America, Western Europe, and Australia, whereas evidence from LMICs remains limited. This imbalance may reduce the generalizability of findings because healthcare infrastructure, workforce capacity, cultural attitudes toward mental health, and access to services differ substantially across settings. Furthermore, interventions developed and evaluated in high-income countries may require adaptation before implementation in LMICs. Resource constraints, shortages of mental health specialists, limited availability of referral services, and differences in healthcare organization may affect both feasibility and effectiveness. Approaches such as task-sharing, integration of mental health services into existing primary healthcare structures, community-based support, and the use of scalable digital technologies may represent promising strategies for adapting suicide prevention interventions to resource-constrained settings. Additional implementation research in LMICs is needed to identify context-specific models that are both feasible and sustainable.

Second, many studies focus on intermediate outcomes such as suicidal ideation, depression, psychological distress, treatment engagement, or healthcare utilization rather than suicide attempts and suicide mortality. Although several interventions demonstrated improvements in these intermediate outcomes, evidence for reductions in suicide attempts and especially suicide mortality remains limited and inconsistent across intervention types. Given the relatively low frequency of suicide deaths, large sample sizes and long follow-up periods are often required to demonstrate intervention effects on suicide-specific outcomes. Consequently, the effectiveness of many PHC-based interventions in reducing suicide mortality remains uncertain and warrants further investigation.

Third, socially vulnerable populations remain underrepresented in intervention research. Although groups such as homeless individuals, migrants, survivors of domestic violence, people with disabilities, and marginalized communities experience disproportionately high suicide risk, relatively few studies have evaluated interventions specifically designed for these populations. Furthermore, vulnerable groups are frequently excluded from clinical trials because of complex social and health-related needs.

Another important limitation is the lack of implementation research. While many interventions have demonstrated efficacy under controlled conditions, fewer studies have examined long-term sustainability, scalability, cost-effectiveness, and integration into routine primary healthcare practice. Understanding how interventions can be successfully adapted to different healthcare systems and resource settings remains an important research priority.

Finally, limited evidence exists regarding multicomponent approaches that simultaneously address both mental health needs and social determinants of suicide risk. Future studies should explore integrated models that combine clinical interventions with strategies targeting poverty, housing instability, unemployment, social isolation, discrimination, and barriers to healthcare access, particularly among socially vulnerable populations.

#### 3.9.2 Limitations of the review

This review has several limitations. First, as a narrative review, it incorporated evidence from a heterogeneous range of sources, including primary studies, systematic reviews, meta-analyses, and narrative reviews. Consequently, the strength of evidence varies across intervention categories and vulnerable populations. While some conclusions are supported by high-level evidence, such as systematic reviews and meta-analyses, other findings are derived primarily from individual studies and should be interpreted with caution. Second, a formal risk-of-bias assessment was not performed because of the narrative design of the review. Finally, despite efforts to provide a comprehensive overview, some relevant studies may not have been identified.

## 4 Conclusion

Suicide prevention in primary health care requires integrated, multidisciplinary approaches that address both mental health needs and the broader social determinants of suicide risk. Across socially vulnerable populations, common factors such as poverty, social exclusion, stigma, unstable housing, limited social support, and barriers to healthcare access contribute to increased vulnerability and often coexist, creating cumulative risk. The evidence reviewed suggests that interventions are most effective when implemented as part of coordinated systems of care that promote continuity, collaboration between health and social services, and timely access to appropriate support.

However, the strength of evidence varies across intervention types. While many interventions have demonstrated improvements in intermediate outcomes, including suicidal ideation, treatment engagement, and healthcare utilization, evidence for reductions in suicide attempts and particularly suicide mortality remains limited. Furthermore, most available evidence originates from high-income countries, raising questions about the transferability and sustainability of interventions in resource-constrained settings.

Future research should prioritize implementation studies, evaluation of integrated models of care, and adaptation of suicide prevention strategies to low- and middle-income countries. Particular attention should be given to interventions that simultaneously address clinical needs and social vulnerabilities in order to reduce inequities in suicide risk and improve outcomes among socially vulnerable populations.
